# Rogue-Elephant-Inflicted Panfacial Injuries: A Rare Case Report

**DOI:** 10.1155/2012/127957

**Published:** 2012-11-11

**Authors:** Santosh Kumar Yadav, Suraksha Shrestha, Srijana Mishra Sapkota

**Affiliations:** ^1^Department of Oral and Maxillofacial Surgery, Chitwan Medical College Teaching Hospital, Chitwan, P.O. Box-42, Bharatpur 10, Nepal; ^2^Department of Prosthodontics, National Academy of Medical Sciences, Bir Hospital, P.O. Box-13606, Kathmandu, Nepal

## Abstract

Attacks by elephants, the largest of the “large animals,” produce many fatalities a year. Most attacks are provoked, although rogue elephants are occasionally responsible. Trampling, goring, tossing the individual with the trunk, or crushing with the knees produces the injuries. Injuries from encounters with large animals represent a significant health risk for rural communities. Wild-animal-inflicted maxillofacial injuries are rare, and limited literature is available describing their management. We present a case of severe maxillofacial injuries caused by the attack of a rogue elephant.

## 1. Introduction

Wild-animal-inflicted injuries are known to be common in rural populations. Strict conservation of wildlife and encroachment into habitat have led to an increase in the number of wild-animal-inflicted injuries and fatalities. Although elephant-human encounter is rare, it can induce serious bone and soft tissue damages leading to aesthetic deformities and functional disabilities. We report a case of a 22-year-old male who sustained maxillofacial injuries inflicted by a rogue elephant.

## 2. Case Report

A 22-year-old male presented with severe maxillofacial injuries caused by a rogue elephant. He encountered the rogue elephant when he was driving his bike outside the village. He was caught by the elephant by its trunk, flung off the bike, and stamped on the face resulting in multiple facial bone fractures and serious soft tissue damage. The patient was admitted in a local hospital where primary care was given and referred to our centre on the third day.

**Figure 1:**
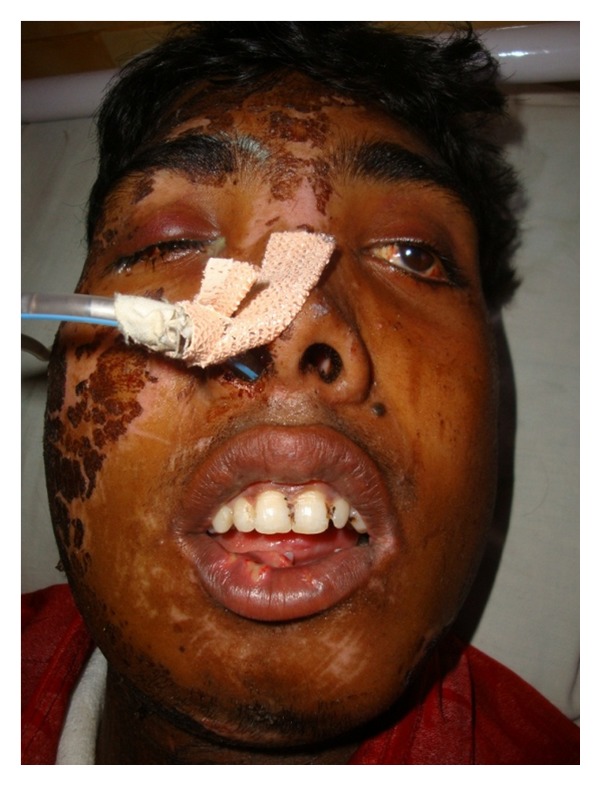
Preoperative.

**Figure 2:**
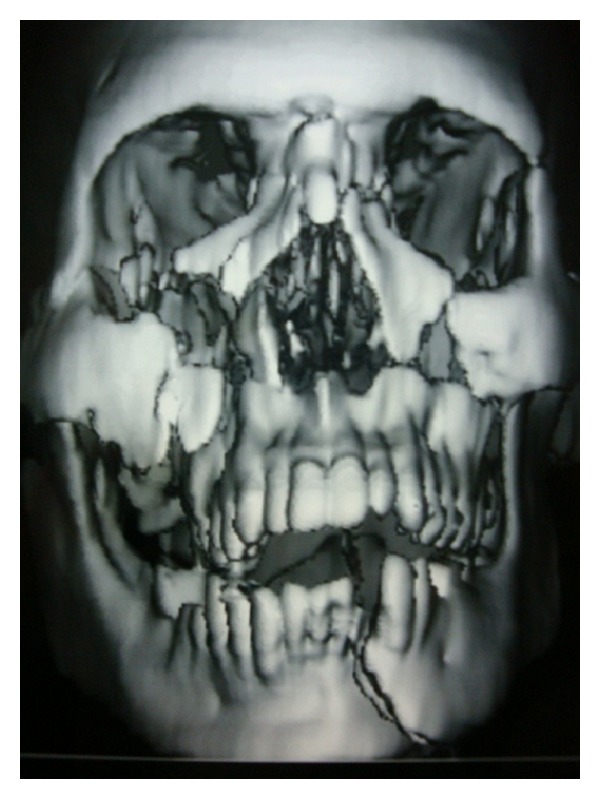
Three-dimensional computed tomography scan showing multiple fractures of jaw bone.

On arrival, his vitals were stable. He was fully conscious, well oriented with time, place, and person. Computed tomography scan was advised, and neurological complications were ruled out. Computed tomography images revealed mandibular left parasymphysis fracture, bilateral Lefort II fractures with comminuted anterior, medial and posterior walls of maxilla (Figures 1 and 2). The right zygoma was downwardly rotated and displaced, and bilateral infraorbital rim fractures were present.

**Figure 3:**
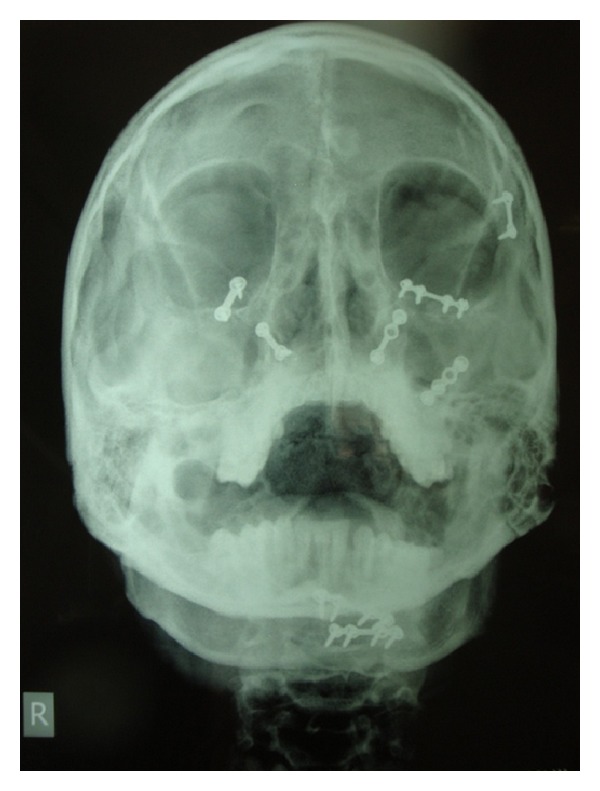
Postoperative paranasal sinus view after 1 year.

**Figure 4:**
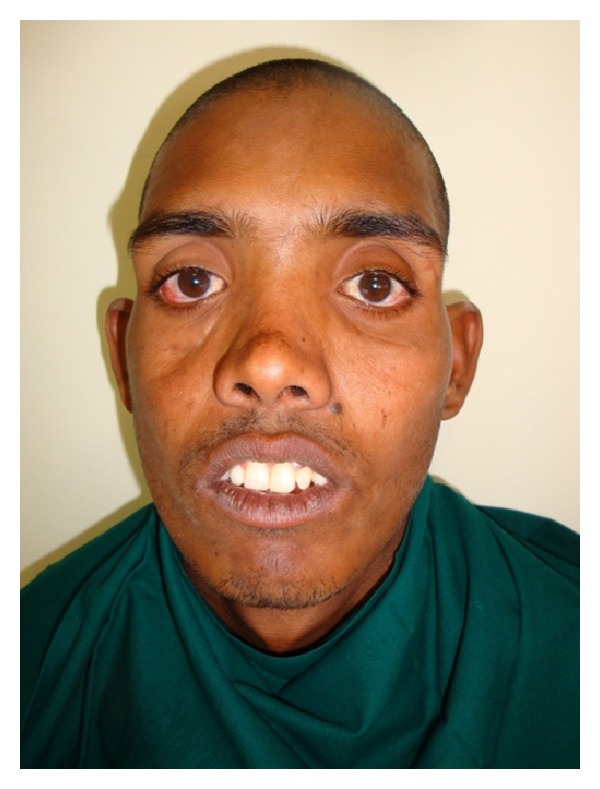
Postoperative 1 year.

Open reduction and rigid internal fixation of the maxillofacial fractures were achieved by using miniplates osteosynthesis under general anesthesia by submental intubation. Postoperative healing was uneventful. Aesthetically pleasing and functionally acceptable result was obtained. On one-year followup, there was no evidence of any complications (Figures 3 and 4).

## 3. Discussion

Wild animal inflicted maxillofacial injuries are rare. However, recent decrease in forest area has increased the chances of elephant-human interaction, hence causing injuries to humans. These attacks take place in remote areas in the vicinity of dense forests, with substantial delay before notification and rescue. Since health-care facilities are deficient in these regions, several injured victims have to be transported hundreds of kilometers, and most die before receiving definitive care. Several factors need to be considered when evaluating these injuries, including type of animal involved, specific nature and location of wounds, and circumstances of attack and interval between injury and treatment. Of greatest concern are direct destruction of tissue and risk of infection [1]. There should be a high index of suspicion when treating these casualties, as serious underlying bone or soft-tissue damage can be overlooked [2].

Injuries by elephants differ from other animals due to the severity of the impact. The gross facial deformity, the larger area of body involved, and the obvious life-threatening complications make it a more difficult entity to treat.

Treatment of life threatening injuries come first, and the serious psychological trauma to victims is usually neglected. A search of the published literature does not reveal much about the nature and management of animal-inflicted injuries [3, 4]. Apart from severe lacerations and penetrating wounds to the head and neck, the victim commonly sustains comminuted fractures of the first and second cervical vertebrae with resultant high laceration of the spinal cord. Major vascular injury and trauma to the pharynx may further compound the problem [3], and without urgent treatment, death may rapidly ensue.

The management of these injuries should start at the site of attack, with measures to stop bleeding, fluid and blood transfusion, and immediate transport to a trauma centre. A detailed examination of the whole body should always be carried out. A well-coordinated team, including neurosurgeon, orthopedic surgeon, maxillofacial surgeon, ophthalmologist, and psychiatrist, should be organized for the management of these cases. Survivors should routinely undergo radiography of the skull, cervical spine, chest, and upper limbs. Computed tomography of the skull and maxillofacial regions helps in formulating the treatment plan.

All victims of an animal attack should be considered to have major trauma, and stabilization of the victim remains the prime objective. After initial resuscitation, management consists of standard wound treatment and copious irrigation with debridement of devitalized tissue [5]. Administration of antibiotics should be routine and of tetanus prophylaxis in nonimmunized cases. The fractured bones need stabilization by means of wiring or plating. Timely intervention, generous wound debridement, and meticulous soft tissue handling including skin approximation can be functionally and aesthetically gratifying. Outcomes that are more fruitful could be determined by the skill of the available medical personne l, medical equipment, and the location in which the victim is first received [6].

Once the patient is stabilized, reconstruction is an arduous task involving meticulous step-by-step reconstruction of the traumatic site into its former self. Mostly, it involves the extensive, but many a time futile, search for the stable area from which the support and fixation can be done. It requires sound anatomical knowledge coupled with a good amount of creativity and imagination of the facial structures, especially in patients in whom the pretrauma pictures are not available.

For management of patients with severe maxillofacial injuries caused by wild animals, a similar approach to that of patients with multiple injuries caused by motor vehicle accidents should be employed. Meticulous examination, life support measures, and relevant surgical procedures should be instituted immediately to minimize morbidity and mortality arising from these injuries.
